# Multicenter Assessment of Postoperative Mastocheck^®^ Dynamics Following Curative Breast Cancer Surgery

**DOI:** 10.3390/cancers18060986

**Published:** 2026-03-18

**Authors:** Hyukjai Shin, Yumi Kim, Jung Min Park, Chan Seok Yoon, Sung-Soo Kim, Dong-Young Noh

**Affiliations:** 1 Breast and Thyroid Surgery, Department of Surgery, Myongji Hospital, Hanyang University Medical Center, Goyang-si 10475, Republic of Korea; drgsshj@mjh.or.kr; 2 Department of Surgery, CHA Gangnam Medical Center, CHA University School of Medicine, Seoul 06062, Republic of Korea; ymkim@chamc.co.kr (Y.K.); minnyang26@chamc.co.kr (J.M.P.); yoondoc@chamc.co.kr (C.S.Y.); 3 Bertis Inc., Gwacheon-si 13840, Republic of Korea

**Keywords:** breast cancer, serum biomarkers, clinical proteomics, postoperative monitoring, multicenter study

## Abstract

Purpose: Previous single-institution studies reported postoperative decreases in Mastocheck^®^ values following breast cancer surgery. This study aimed to assess the reproducibility of these postoperative changes using two independent clinical cohorts with distinct postoperative assessment designs. Methods: Postoperative Mastocheck^®^ dynamics were evaluated in two independent cohorts with distinct postoperative assessment designs. In the CHA Gangnam Medical Center (CHA University/CHA Medical Center, Seoul, Republic of Korea) cohort, Mastocheck^®^ values obtained before surgery were compared with postoperative values preoperative values (*n* = 210) were compared with an independent postoperative set obtained ≥6 months after surgery (*n* = 171; median 378 days), and a paired subset (*n* = 28) was analyzed for within-patient changes. In an independent cohort from Myongji Hospital, only postoperative patients were enrolled, and longitudinal Mastocheck^®^ measurements were analyzed in patients with four consecutive postoperative assessments. Changes in Mastocheck^®^ values and transitions across the predefined diagnostic cut-off (0.0668) were evaluated. Results: In the CHA cohort, Mastocheck^®^ values were significantly lower in the postoperative set than in the preoperative set (*p* < 0.001). Within the paired subset, among patients above the cut-off preoperatively (*n* = 20), 90.0% (18/20) converted to below the cut-off postoperatively, and paired analysis confirmed a significant postoperative decrease (Wilcoxon signed-rank test, two-tailed, *p* = 1.72 × 10^−4^). In the Myongji cohort, longitudinal follow-up demonstrated a sustained decline in Mastocheck^®^ values over time, accompanied by a stepwise reduction in the proportion of patients exceeding the cut-off, reaching 0% at the final follow-up. Conclusions: Across two independent cohorts with complementary postoperative assessment strategies, Mastocheck^®^ values consistently decreased after breast cancer surgery and shifted to, or were maintained below, the predefined diagnostic cut-off. These findings reproduce and extend prior single-institution observations, supporting the reproducibility of Mastocheck^®^ postoperative decline dynamics and cut-off transitions across independent cohorts. This study did not evaluate recurrence detection, imaging concordance, or survival outcomes, and therefore does not establish clinical surveillance performance.

## Simple Summary

These reproducible postoperative changes support Mastocheck® as a complementary blood-based readout for characterizing postoperative decline dynamics and contextualizing follow-up measurements. In this study, consistent decreases in Mastocheck® values were observed across two independent cohorts with different postoperative assessment designs, along with transitions from above to below the predefined diagnostic cut-off after surgery. These findings indicate stable normalization of tumor-associated signals over time and provide a useful framework for interpreting longitudinal measurements during follow-up. However, surveillance performance for recurrence detection was not evaluated and requires validation in future outcome-adjudicated prospective studies.

## 1. Introduction

Breast cancer is the most commonly diagnosed malignancy among women worldwide, and advances in early detection and treatment have led to steadily improving survival outcomes [1,2]. Consequently, the number of breast cancer survivors requiring long-term postoperative surveillance has increased substantially. Current follow-up strategies rely primarily on history taking, physical examination, and imaging modalities such as mammography, with or without adjunctive ultrasonography or magnetic resonance imaging [3,4]. However, these approaches have inherent limitations, particularly in women with dense breast tissue [5,6] and in the postoperative breast, where benign post-treatment changes can overlap with tumor recurrence and complicate image interpretation [7]. In this context, there is growing clinical demand for objective and reproducible blood-based tools that can complement conventional imaging-based postoperative surveillance.

Blood-based biomarkers for breast cancer have traditionally focused on diagnostic or prognostic applications. However, most conventional serum markers, including CA15-3, show limited sensitivity and specificity in early-stage disease and provide restricted clinical utility for routine postoperative monitoring [8,9].

In contrast, proteomics-based assays enable quantitative and reproducible assessment of multiple tumor-associated proteins and have become clinically applicable platforms rather than exploratory research tools [10,11]. Mastocheck^®^, a three-protein signature assay based on multiple reaction monitoring mass spectrometry, was developed as a blood-based adjunct for breast cancer diagnosis and received regulatory approval as a class III in vitro diagnostic medical software (MFDS approval No. 19-5; model: BM_1BCM) by the Korean Ministry of Food and Drug Safety in January 2019 [12,13,14]. Accordingly, Mastocheck^®^ represents an approved diagnostic support tool with established analytical and clinical validity.

Beyond its approved role in aiding primary diagnosis, subsequent clinical studies have consistently demonstrated that Mastocheck^®^ values exhibit a postoperative downward shift following curative breast cancer surgery. This dynamic change has drawn attention to its potential applicability in postoperative follow-up settings. A prior longitudinal study conducted at a single tertiary referral center reported a significant postoperative downward shift in Mastocheck^®^ values over time, with many patients showing sustained decreases during follow-up, suggesting that the assay reflects changes associated with tumor burden reduction rather than static diagnostic classification alone [15]. While these findings were clinically encouraging, their interpretation was inherently constrained by the single-institution design.

Reproducibility across independent cohorts represents a critical requirement for biomarkers intended for serial monitoring rather than one-time diagnostic assessment [16,17]. Postoperative breast cancer patients constitute a clinically distinct population in whom biomarker utility depends not only on diagnostic accuracy but also on the consistent and interpretable temporal behavior of biomarker values across repeated measurements [18]. Without external validation, postoperative changes observed in a single institution may reflect center-specific patient selection, perioperative management, or analytical factors, thereby limiting generalizability.

Therefore, the present study was designed to evaluate whether the postoperative downward shift in Mastocheck^®^ values previously reported can be reproduced across independent clinical settings. Using multicenter cohorts collected in different institutional environments with distinct patient flow and follow-up practices, we aimed to access the reproducibility and clinical interpretability of postoperative Mastocheck^®^ decline dynamics following breast cancer surgery. Importantly, this study focuses on postoperative kinetics and cut-off transitions rather than clinical surveillance endpoints; recurrence detection, imaging concordance, and survival outcomes were not assessed and should be addressed in future outcome-adjudicated prospective studies.

From a broader clinical perspective, despite advances in systemic therapy and surgical techniques, a substantial proportion of patients remain at risk of locoregional or distant recurrence during long-term follow-up, particularly in specific molecular subtypes and younger populations [19,20,21]. The goal of postoperative surveillance is not merely to detect recurrence, but to detect it at a stage when therapeutic intervention may meaningfully improve outcomes. Current international guidelines recommend periodic history taking, physical examination, and annual mammography, while discouraging routine laboratory testing or advanced imaging in asymptomatic patients [22,23,24]. However, in real-world practice, concerns persist regarding the limitations of symptom-driven follow-up strategies [25], particularly in younger women and those with dense breasts, where conventional imaging performance can be limited and supplemental imaging strategies are actively being evaluated [5,6,26]. Furthermore, postoperative architectural distortion, fibrosis, and other treatment-related changes can obscure radiologic interpretation and increase diagnostic uncertainty [7,27].

In this setting, there is increasing interest in blood-based biomarkers—including circulating proteins and emerging liquid biopsy approaches such as circulating tumor DNA—that reflect biological tumor burden and systemic tumor–host responses rather than structural abnormalities alone [11,28,29]. Traditional serum tumor markers such as CA15-3 have been used in clinical practice, but their sensitivity for early-stage disease and utility for routine surveillance are limited [8,9]. Beyond conventional tumor markers, inflammatory mediators—including cytokines and chemokines involved in immune modulation and tumor–microenvironment crosstalk—have been linked to breast cancer development and progression [30]. An ideal postoperative biomarker should therefore demonstrate not only diagnostic discrimination, but also a consistent directional response to tumor removal and temporal stability in the absence of recurrence [15,16]. Such dynamic behavior would enhance clinical interpretability and allow biomarker trends to complement imaging findings during surveillance. For this reason, demonstrating reproducible biomarker kinetics across independent clinical environments is an important step toward broader evaluation of postoperative monitoring strategies, and should ideally be followed by prospective studies with standardized sampling and outcome-linked follow-up [15,16].

## 2. Methods

### 2.1. Study Design and Patients

This multicenter observational study evaluated perioperative and postoperative changes in Mastocheck^®^ values in patients with pathologically confirmed breast cancer.

Patients were recruited from CHA Gangnam Medical Center and Myongji Hospital between June 2022 and July 2025. At CHA Gangnam Medical Center, patients were prospectively enrolled and followed according to a predefined study protocol. In contrast, data from Myongji Hospital were retrospectively analyzed using previously obtained clinical Mastocheck^®^ test results, following Institutional Review Board approval for secondary data use.

**Figure 1:**
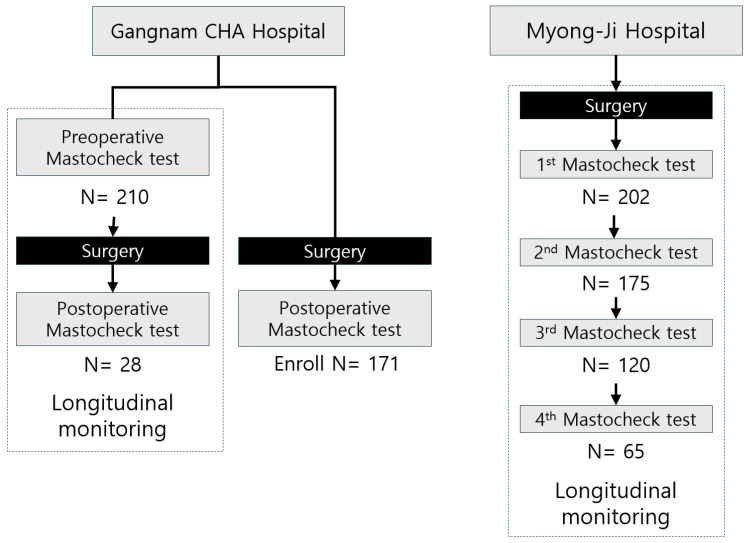
Study design and Mastocheck^®^ testing timeline at two independent institutions. At CHA Gangnam Medical Center, patients with pathologically confirmed breast cancer underwent Mastocheck^®^ testing before surgery, followed by surgical treatment, and a subset received an additional Mastocheck^®^ test at least 6 months after surgery. At Myongji Hospital, postoperative breast cancer patients were enrolled and underwent serial Mastocheck^®^ testing during follow-up, with a subset completing four consecutive postoperative measurements. This figure illustrates the different perioperative and postoperative testing schemes used to evaluate longitudinal changes in Mastocheck^®^ values across the two study cohorts.

Although enrollment differed by site (prospective at CHA vs. retrospective secondary analysis at Myongji), the study evaluated the same endpoint in both cohorts using the identical diagnostic cut-off and prespecified timepoint definitions. The overall study design and institution-specific Mastocheck® testing schedules are shown in Figure 1. To assess potential between-cohort heterogeneity, we conducted a cohort-stratified sensitivity analysis (Supplementary Figure S1) to assess whether the postoperative trend was consistent across institutions.

### 2.2. Study Area

This multicenter study was conducted in breast cancer care centers at Myongji Hospital and CHA Gangnam Medical Center (Republic of Korea). Eligible participants were managed according to institutional clinical pathways, and blood samples for Mastocheck^®^ analysis were obtained and processed under harmonized study procedures.

### 2.3. CHA Gangnam Medical Center Cohort

At CHA Gangnam Medical Center, 210 patients with histologically confirmed breast cancer underwent a preoperative Mastocheck^®^ test prior to definitive surgery. All patients subsequently underwent curative breast cancer surgery. Among these, 28 patients had paired pre- and postoperative measurements, which were used for within-patient comparisons of postoperative changes. In addition, postoperative samples (≥6 months after surgery) obtained during routine follow-up were available as a separate postoperative dataset at the same institution (*n* = 171).

Postoperative assessments were defined using prespecified time windows relative to surgery (Post1: >6 months after surgery; Post2–Post4: approximately 6-month intervals thereafter).

### 2.4. Myongji Hospital Cohort

At Myongji Hospital, only patients who had already undergone breast cancer surgery were included. Between June 2022 and July 2025, all postoperative breast cancer patients within 5 years after surgery who had previously provided consent for the secondary use of their clinical data were included, and a total of 202 patients underwent serial Mastocheck^®^ testing as part of routine postoperative follow-up.

Among these, 68 patients had four consecutive postoperative measurements; after exclusion of three patients with a postoperative interval exceeding 5 years, 65 patients were included in the longitudinal analysis.

Accordingly, the longitudinal (four-successive-measure) analysis in the retrospective Myongji cohort was restricted to patients with complete serial testing within prespecified time windows. Patients lacking any required timepoint—due to missed follow-up, testing outside the predefined windows, follow-up at another facility, or discontinuation of additional postoperative testing—were not included in the four-timepoint analysis. Because this study was embedded in routine postoperative care, continued serial testing depended on voluntary attendance at scheduled follow-up assessments and could not be mandated; therefore, attrition over time was expected in real-world follow-up. We acknowledge that restricting the longitudinal analysis to complete cases may introduce residual selection bias related to follow-up completeness.

### 2.5. Ethics Approval

The study was conducted in accordance with the Declaration of Helsinki and approved by the Institutional Review Board of CHA Gangnam Medical Center (GCI IRB 2022-04-004). Secondary use of retrospective clinical Mastocheck^®^ data from Myongji Hospital was approved by the Institutional Review Board of Myongji Hospital (MJH IRB 2025-11-014; approved November 2025). Written informed consent was obtained from all participants in the prospective CHA cohort. For the retrospective Myongji cohort, the requirement for informed consent was waived by the IRB due to the use of existing clinical data.

### 2.6. Eligibility and Data Screening

Women aged ≥19 years who provided written informed consent were eligible. For the breast cancer group, we included patients with pathologically confirmed breast cancer (stage 0–IV) who provided a pre-treatment blood sample (before surgery or neoadjuvant chemotherapy) and agreed to paired post-treatment sampling (after surgery and/or neoadjuvant chemotherapy, as applicable). We excluded patients with recurrent breast cancer, those diagnosed with another malignancy within the past 5 years, those in whom another malignancy was identified during metastatic work-up, and those who had initiated breast cancer treatment before baseline sampling; patients deemed unsuitable by the investigator were also excluded. For the surveillance/follow-up group, we included women undergoing routine follow-up in the breast clinic with consent and excluded those who had already started breast cancer treatment, had evidence of another malignancy on metastatic evaluation, or were considered unsuitable by the investigator.

### 2.7. Mastocheck^®^ Analysis

Mastocheck^®^ is a blood-based proteomic assay that quantifies three plasma proteins—apolipoprotein C-1 (APOC1), carbonic anhydrase 1 (CAH1), and neural cell adhesion molecule L1-like protein (NCHL1)—using multiple reaction monitoring–based liquid chromatography–tandem mass spectrometry (MRM LC–MS/MS; SCIEX, Framingham, MA, USA). Plasma sample pretreatment, including protein denaturation, reduction, alkylation, enzymatic digestion, and peptide cleanup, was performed according to a previously described and validated protocol [31]. Quantitative analysis was conducted using stable isotope-labeled internal standards, and the composite Mastocheck^®^ score was calculated using a previously validated algorithm. A predefined cut-off value was applied to classify results as within the cancer or non-cancer range. Across both institutions, Mastocheck^®^ values were generated using the same three-protein algorithm and the same prespecified diagnostic cut-off. Sample handling and LC–MS/MRM processing followed an identical SOP and quality-control criteria applied throughout the study period. Eligibility criteria and definitions of each postoperative timepoint were harmonized between the prospective and retrospective cohorts.

All Mastocheck^®^ measurements were performed using an identical standardized protocol and a predefined algorithm. Samples were de-identified and analyzed under coded identifiers, and laboratory personnel were not involved in clinical care or follow-up assessments. Batch-level quality control procedures were applied, including the use of internal standards and predefined acceptance criteria, with repeat measurement performed when QC criteria were not met. All assays were performed at a single central laboratory (Mastocheck^®^ Center, Bertis Inc., Yongin-si, Republic of Korea) using the same SOP across cohorts. Samples were de-identified and analyzed under coded identifiers, and laboratory personnel were blinded to clinical information and follow-up timepoint.

### 2.8. Statistical Analysis

Mastocheck^®^ values were summarized as median (interquartile range, IQR) and visualized using distribution plots. The diagnostic cut-off (0.0668) was prespecified according to prior validation of the Mastocheck^®^ three-protein algorithm and was applied unchanged in the present study. For between-group comparisons in the unpaired analysis (independent preoperative vs. postoperative cancer samples, including routine follow-up samples without matched preoperative measurements), the Mann–Whitney U test was used. For within-patient comparisons in the paired cohort (pre-operation vs. post-operation), the Wilcoxon matched-pairs signed-rank test was applied. A two-tailed test was used for the paired analysis, with a prespecified expected postoperative decrease (pre-operation > post-operation); all other tests were two-sided unless stated otherwise. Statistical significance was defined as *p* < 0.05. For comparisons of proportions, the χ^2^ test was used. Analyses were implemented in Python (version 3.11.7) using SciPy (version 1.11.4).

## 3. Results

### 3.1. Patient Characteristics

Baseline characteristics of the study cohorts are summarized in Table 1, including age, stage distribution, molecular subtype, and treatment exposure. The cohorts showed differences in clinical composition, which should be considered when interpreting cohort-level comparisons. The mean age was comparable between the CHA cohort (52.27 ± 10.45 years, *n* = 210) and the Myongji cohort (53.48 ± 8.76 years, *n* = 202). Although distributions of tumor stage and nodal status differed between the cohorts, with a higher proportion of T0 disease in the CHA cohort and a greater representation of T1 tumors and advanced nodal disease in the Myongji cohort, early-stage disease predominated in both populations. Subtype distributions, assessed in patients with invasive breast cancer only, were similar overall, with luminal subtypes accounting for the majority of cases.

### 3.2. Postoperative Changes in Mastocheck^®^ Values (CHA Cohort)

We first evaluated within-patient postoperative change in the paired subset with available pre- and postoperative measurements (*n* = 28; median postoperative interval, 378 days), to directly assess postoperative decline independent of between-group differences. We then examined the broader unpaired comparison between preoperative samples (*n* = 210) and an independent postoperative follow-up set obtained ≥6 months after surgery (*n* = 171) as supportive evidence of a cohort-level distributional shift.

**Figure 2:**
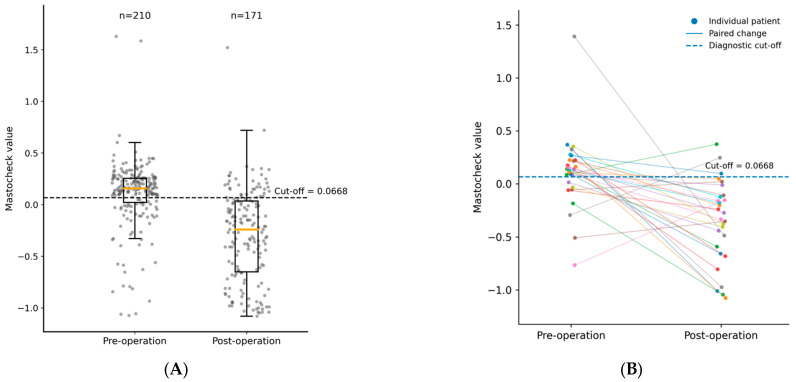
Individual changes in Mastocheck values before and after surgery with diagnostic cut-off. (**A**) Distribution of Mastocheck^®^ values in the pre-operation and post-operation cancer groups. Horizontal yellow solid bars indicate median values. The black dashed line denotes the predefined diagnostic cut-off (0.0668). (**B**) Individual paired changes in Mastocheck^®^ values before and after surgery in patients with available paired measurements (*n* = 28). Each dot represents an individual patient, and paired measurements are connected by lines. Among patients with preoperative Mastocheck^®^ values above the cut-off, 90.0% (18/20) converted to values below the cut-off after surgery. Mastocheck^®^ values significantly decreased after surgery (Wilcoxon matched-pairs signed-rank test, two-tailed [Pre > Post], *p* = 1.72 × 10^−4^).

In the paired subset with available pre- and postoperative measurements (*n* = 28; median postoperative interval, 378 days), Mastocheck^®^ values also decreased significantly after surgery (Wilcoxon matched-pairs signed-rank test, two-tailed, *p* = 1.72 × 10^−4^), with most individual trajectories crossing below the diagnostic cut-off (Figure 2). Within the paired subset, 20 patients were above the diagnostic cut-off preoperatively, and 18 of these (90.0%) converted to below the cut-off postoperatively. Within the paired subset, the distribution also showed a marked downward shift (Supplementary Figure S2), with the median Mastocheck^®^ value decreasing from 0.134 preoperatively to −0.304 postoperatively (Wilcoxon signed-rank test, two-tailed, *p* = 1.72 × 10^−4^).

In the unpaired analysis, Mastocheck^®^ values were significantly lower in the postoperative group (*n* = 171) than in the preoperative group (*n* = 210) (Mann–Whitney U test, two-tailed, *p* < 0.001). Consistent with this distributional shift, the proportion of subjects with Mastocheck^®^ values above the diagnostic cut-off was higher in the preoperative group (72.8%, 153/210) than in the postoperative group (22.2%, 38/171) (χ^2^ test, *p* < 0.001; Supplementary Figure S1).

### 3.3. Longitudinal Postoperative Follow-Up (Myongji Hospital Cohort)

**Figure 3:**
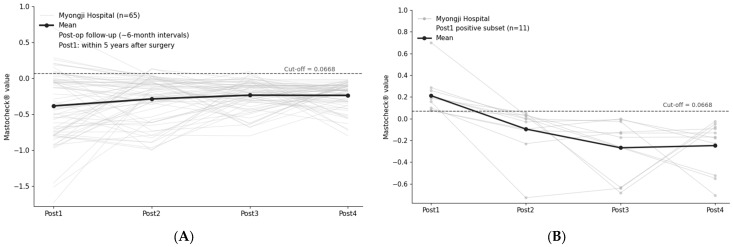
Longitudinal Mastocheck^®^ trajectories in the complete-case Myongji cohort (*n* = 65) and the subset positive at Post1 (*n* = 11). (**A**) Individual Mastocheck^®^ values across four postoperative time points (Post1–Post4) in the complete-case longitudinal cohort (*n* = 65). The dashed horizontal line indicates the diagnostic cut-off (0.0668). Post1(first postoperative assessment) represents the first postoperative assessment within 5 years after surgery, followed by serial assessments at approximately 6-month intervals. (**B**) Longitudinal trajectories of the subset of patients who were positive at Post1 (Mastocheck^®^ ≥ 0.0668) within the complete-case–cohort (*n* = 11 of 65). All 11 patients shifted below the diagnostic cut-off from Post2 onward, and values remained below the cut-off through Post3 and Post4. The dashed horizontal line again indicates the diagnostic cut-off (0.0668).

Consistent with the postoperative decrease observed in the CHA cohort (Figure 2), longitudinal follow-up in Myongji Hospital cohort demonstrated that Mastocheck^®^ values remained below the diagnostic cut-off during postoperative follow-up (Figure 3).

Longitudinal postoperative follow-up of Mastocheck^®^ values was evaluated in Myongji Hospital (*n* = 65). Post1(first postoperative assessment) was defined as the first postoperative assessment performed within 5 years after surgery (median interval, 545 days), followed by three additional assessments (Post2–Post4) conducted at approximately 6-month intervals.

In the overall cohort, Mastocheck^®^ values were predominantly maintained below the diagnostic cut-off of 0.0668 after surgery, and this below–cut-off distribution was consistently preserved across subsequent longitudinal measurements (Figure 3A).

In a sensitivity analysis using all available samples at each postoperative assessment (available-case), positivity decreased monotonically across Post1–Post4 despite attrition (34/202, 3/175, 1/120, and 0/65; Supplementary Figure S1).

### 3.4. Subgroup Analysis of Patients with Elevated Values at Post1

To further characterize postoperative trajectories among patients with initially elevated values, we analyzed the subset of patients with Mastocheck^®^ values ≥ 0.0668 at Post1 within the longitudinal cohort with complete follow-up through Post4 (*n* = 65). In this complete-case longitudinal cohort, 11 patients were positive at Post1 (11/65, 16.9%) (Figure 3B).

Among these patients, from Post2 onward, all 11 patients shifted below the diagnostic cut-off, and no patients remained positive at Post2, Post3, or Post4 (0/65 at each timepoint). These findings indicate that transient postoperative elevations at Post1 were followed by sustained values below the diagnostic cut-off during subsequent follow-up.

For clarity, this subset was defined solely by Post1 positivity within the complete-case longitudinal cohort (*n* = 65) and was not selected based on subsequent trajectories.

## 4. Discussion

The present study was designed to assess the reproducibility of postoperative changes in Mastocheck^®^ values across independent clinical settings. To this end, we analyzed data obtained from two independent multicenter cohorts, encompassing distinct postoperative evaluation structures. Despite differences in patient flow and follow-up practices between centers, consistent postoperative patterns in Mastocheck^®^ values were observed across both cohorts, supporting inter-cohort consistency of postoperative dynamics.

Our findings align with prior clinical studies of the three-protein signature/Mastocheck^®^, which established diagnostic performance using a predefined cut-off in independent cohorts [13] and in a multicenter prospective trial of women with suspicious lesions [14]. Consistent with a prior follow-up study after breast cancer surgery [15], we observed that postoperative values tend to shift below the diagnostic cut-off over time, supporting interpretation in a time-from-surgery and treatment-context framework. In this context, the present study extends these observations by demonstrating reproducible postoperative cut-off transitions across two institutions with different data sources (prospective vs. retrospective) and follow-up structures, supporting the consistency of this postoperative pattern under real-world clinical workflows.

The observed postoperative decrease in Mastocheck^®^ values is biologically consistent with reduced tumor burden after surgical removal and subsequent attenuation of tumor-associated systemic signals. Because Mastocheck^®^ integrates multiple circulating proteins into a single score, the postoperative trajectory may reflect both direct reduction in tumor-associated protein dysregulation and gradual normalization of host responses (including inflammatory and metabolic perturbations). Notably, early postoperative measurements may additionally be influenced by transient perioperative inflammation and recovery, whereas later time points may capture stabilization under adjuvant systemic therapies. These factors support interpreting Mastocheck^®^ dynamics primarily in a time-from-surgery framework and underscore the need for treatment-aligned sampling in future prospective routine postoperative follow-up studies.

In the CHA Gangnam Medical Center cohort, we evaluated postoperative change using both a broader unpaired comparison and a paired within-patient analysis. The unpaired analysis compared the preoperative set (*n* = 210) with an independent postoperative follow-up set obtained ≥6 months after surgery during routine follow-up (*n* = 171; median postoperative interval, 378 days) and demonstrated a clear distributional shift in Mastocheck^®^ values toward lower levels, accompanied by a reduction in positivity. Consistently, in the paired subset with available pre- and postoperative measurements (*n* = 28), Mastocheck^®^ values showed a pronounced within-patient decrease, with most individual trajectories crossing below the diagnostic cut-off. In particular, within the paired subset, among patients with preoperative Mastocheck^®^ values above the cut-off (*n* = 20), 90.0% (18/20) shifted to values below the cut-off at the postoperative assessment. The observation of this transition at a delayed postoperative time point supports the interpretation that Mastocheck^®^ reflects sustained postoperative changes associated with tumor removal rather than transient perioperative effects (e.g., anesthesia, inflammation, or surgical stress). Taken together, the concordant findings from the paired and unpaired analyses reinforce the internal consistency of this postoperative pattern.

In parallel, longitudinal analysis of the Myongji Hospital cohort demonstrated consistent postoperative Mastocheck^®^ patterns over extended follow-up. In this cohort, all patients had undergone breast cancer surgery, and Mastocheck^®^ measurements were evaluated as part of routine postoperative monitoring. At the first postoperative assessment (Post1), performed more than six months after surgery, the majority of patients already exhibited Mastocheck^®^ values below the diagnostic cut-off. Subsequent assessments conducted at approximately six-month intervals showed a continued decrease in the proportion of patients exceeding the cut-off, ultimately reaching 0% at the final follow-up. Importantly, patients with values above the cut-off at Post1 subsequently transitioned to and remained below the cut-off during continued follow-up, indicating stable maintenance of sub–cut-off values over time.

Because follow-up duration and sampling structures differed between cohorts—longitudinal assessments in the Myongji cohort versus a single postoperative assessment at a relatively long interval in the CHA cohort (median, 378 days)—differences in observation windows could influence the apparent variability of postoperative Mastocheck^®^ values. In particular, the Myongji cohort provided repeated measurements beginning at >6 months after surgery (Post1) and continuing at approximately 6-month intervals, whereas the CHA cohort primarily reflects a later postoperative snapshot. To avoid over-interpreting between-cohort differences that may arise from non-uniform follow-up, our primary inference focuses on within-patient directional change and clinically interpretable cut-off transitions observed in each cohort independently. Additionally, differences in baseline patient and tumor characteristics between cohorts may also contribute to heterogeneity; therefore, we focused on within-cohort postoperative directional changes rather than direct cross-cohort comparisons.

To assess whether differences in data capture (prospective vs. retrospective) influenced the main trend, we performed a cohort-stratified sensitivity analysis of positivity at the first postoperative assessment (Supplementary Figure S1), which demonstrated a consistent reduction in positivity in both cohorts. Nevertheless, residual selection bias due to differential follow-up completeness and unmeasured heterogeneity cannot be fully excluded. Furthermore, recurrence, progression, and survival outcomes were not uniformly captured across cohorts within the available follow-up structure, precluding robust outcome-linked analyses. Accordingly, prospective studies with standardized event ascertainment and outcome-linked follow-up will be needed to clarify the relationship between postoperative Mastocheck^®^ trajectories and recurrence or survival.

Regarding the potential influence of clinicopathologic factors (stage, subtype) and treatment exposure, the present dataset was not powered for formal stratified or adjusted analyses, particularly given the small postoperative-positive subset. Although we explored whether postoperative changes in Mastocheck^®^ differed according to clinicopathologic factors, including stage and molecular subtype, as well as treatment exposure (surgery alone vs. surgery plus adjuvant therapy), the postoperative decrease and cut-off conversion pattern was qualitatively consistent across these strata; however, subgroup sample sizes were limited, and the study was not powered to detect modest between-group differences. Larger prospective studies with standardized sampling aligned to treatment milestones are warranted to quantify potential effect modification by subtype, stage, or adjuvant therapy.

Importantly, this study was not designed to evaluate clinical surveillance outcomes; recurrence events, imaging concordance, and survival endpoints were not adjudicated, and therefore surveillance performance cannot be inferred from the present data.

In addition, although clinicopathologic characteristics and treatment exposures were described, the present study was not powered for formal stratified or adjusted analyses across stage, nodal status, molecular subtype, and adjuvant therapy. Given the limited numbers within several strata—particularly the small postoperative-positive subset—subgroup estimates would be unstable; therefore, potential effect modification should be evaluated in larger protocolized cohorts with comprehensive treatment-milestone capture.

Finally, the study was conducted at only two centers within a single country, and no formal power calculation was performed; therefore, our conclusions emphasize reproducibility of postoperative dynamics across independent cohorts rather than broad population-level generalizability.

Despite these limitations, a key point we wish to emphasize is that, from a clinical decision-support perspective, distinguishing numerical reduction from cut-off–based interpretation is particularly important. Mastocheck^®^ is intended as a decision-support tool interpreted relative to a predefined diagnostic threshold rather than on absolute value changes alone. Accordingly, postoperative transitions across this threshold provide a clinically actionable and interpretable framework that aligns with how surveillance decisions are typically triggered in routine practice. In the CHA cohort, a clear pre-to-postoperative decrease was accompanied by conversion to below the cut-off in the majority of patients, while the Myongji cohort independently confirmed sustained maintenance of sub–cut-off values during extended follow-up. Together, these findings support both the biological relevance of postoperative Mastocheck^®^ dynamics and the practical utility of cut-off–based interpretation for longitudinal monitoring.

Taken together, the reproducible postoperative decrease observed across cohorts with distinct study designs suggests that Mastocheck^®^ reflects durable postoperative biological changes. By demonstrating concordant postoperative shifts across different clinical workflows, this study reproduces and extends prior single-institution observations and provides additional evidence that Mastocheck^®^ captures postoperative changes beyond its approved diagnostic role. Therefore, future prospective studies with standardized sampling schedules and outcome-linked follow-up will be valuable to define optimal testing intervals, clarify its role in recurrence detection and long-term outcomes, and determine its clinical utility as a complementary tool for postoperative surveillance.

## 5. Conclusions

Across two independent clinical cohorts with distinct postoperative assessment designs, Mastocheck^®^ values showed a consistent downward shift following curative breast cancer surgery. In both cohorts, values tended to shift toward, or remain below, the predefined diagnostic cut-off, supporting the reproducibility and clinical interpretability of postoperative Mastocheck^®^ dynamics.

These findings reproduce and extend prior single-institution observations and provide additional evidence that Mastocheck^®^ captures postoperative changes beyond its approved diagnostic role. Because recurrence events, imaging concordance, and survival outcomes were not adjudicated in this study, clinical surveillance performance cannot be determined from the present data. Accordingly, outcome-adjudicated prospective studies with standardized sampling schedules, including recurrence-confirmed endpoints, are warranted to clarify how postoperative Mastocheck^®^ trajectories relate to clinically meaningful outcomes.

Future studies should further validate Mastocheck^®^ for postoperative monitoring in larger cohorts using fully prospective, protocolized designs with harmonized sampling schedules and standardized follow-up windows. Such designs will help reduce heterogeneity related to non-uniform follow-up intensity, missingness, and differences in data sources. Comprehensive capture of clinicopathologic variables and treatment milestones will be important to enable more definitive assessment of potential effect modification by stage, subtype, and adjuvant therapy. Long-term longitudinal studies incorporating adjudicated recurrence endpoints are warranted to characterize Mastocheck^®^ trajectories in patients who develop locoregional or distant recurrence and to refine clinically interpretable, cut-off–based monitoring strategies for routine follow-up. In parallel, beyond postoperative monitoring, future research should evaluate Mastocheck^®^ as a diagnostic decision-support tool in conjunction with breast ultrasound (and other imaging modalities) in patients with suspicious lesions, with the goal of reducing unnecessary biopsies while improving diagnostic accuracy through integrated, multimodal risk stratification.

## Figures and Tables

**Table 1:** Baseline clinicopathologic characteristics of the study cohorts.

	CHA Cohort	Myongji Cohort
Age (avg ± SD)	(*N* = 210) 52.27 ± 10.45 year	(*N* = 202) 53.48 ± 8.76 year
T stage	(*N* = 210, 100.0%)	(*N* = 202, 100.0%)
0	38 (18.1%)	11 (5.4%)
1	94 (44.8%)	111 (55.0%)
2	74 (35.2%)	73 (36.1%)
3+	4 (1.9%)	7 (3.5%)
N stage	(*N* = 210, 100.0%)	(*N* = 202, 100.0%)
0	148 (70.5%)	141 (69.8)
1	50 (23.8%)	36 (19.3)
2	10 (4.8%)	8 (4.0)
3+	2 (0.9%)	14 (6.9)
Subtype	(*N* = 171, 100.0%)	(*N* = 183, 100.0%)
Luminal A	84 (49.1%)	78 (38.6%)
Luminal B	34 (19.9%)	69 (34.2%)
HER2	30 (17.5%)	18 (8.9%)
TNBC	23 (13.5%)	18 (8.9%)

Values are presented as number (%) unless otherwise indicated. Subtype classification was performed only in patients with invasive breast cancer; therefore, denominators differ from the total cohort size. T, tumor stage; N, nodal stage (pathologic nodal stage, pN); HER2, human epidermal growth factor receptor 2. T stage and N stage were classified according to the AJCC TNM system. T stage indicates primary tumor extent (T0–T3/4), and N stage indicates regional lymph node involvement (N0–N3). “3+” denotes stage ≥3 (e.g., T3–T4 or N3). Molecular subtypes (Luminal A, Luminal B, HER2-enriched, and TNBC) were defined based on routine immunohistochemistry for ER, PR, and HER2.

## Data Availability

The data presented in this study are not publicly available due to privacy and ethical restrictions. De-identified data may be available from the corresponding author upon reasonable request and with permission of the Institutional Review Board.

## Supplementary Materials

The following supporting information can be downloaded at https://www.mdpi.com/article/10.3390/cancers18060986/s1: Supplementary Figure S1: Available-case sensitivity analysis of postoperative positivity across cohorts; Supplementary Figure S2: Distribution of Mastocheck values before and after surgery.
