# Supplementary material for: Multicenter Assessment of Postoperative Mastocheck® Dynamics Following Curative Breast Cancer Surgery
Source: Cancers (Basel). 2026 Mar 18;18(6):986. doi: 10.3390/cancers18060986 (PMC13024769; doi:10.3390/cancers18060986)
Supplement: Supplementary file 1 [file cancers-18-00986-s001.zip › cancers-4150029-supplementary.pdf]

cancers

MDPI

Supplementary Materials: Multicenter Validation of Postoperative Mastocheck® Dynamics Following Breast Cancer Surgery

Hyukjai Shin, Yumi Kim, Sung-Soo Kim, Dong-Young Noh

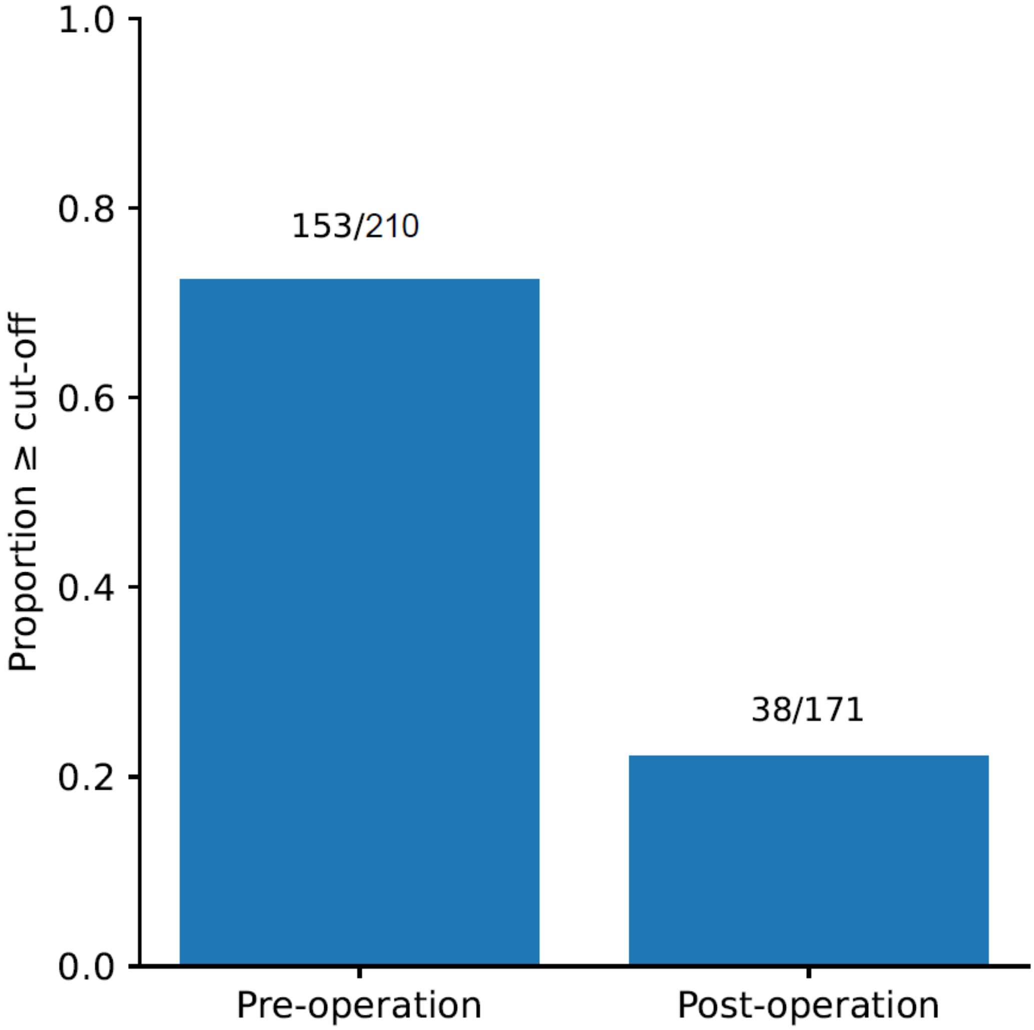

Figure S1. Proportion of subjects with Mastocheck values above the diagnostic cut-off in pre-operation and post-operation cohorts.

Bars indicate the proportion of subjects with Mastocheck values ≥ 0.0668 in each cohort. Error bars represent 95% confidence intervals. The pre-operation group showed a substantially higher proportion of values above the cut-off compared with the post-operation group.

 https://doi.org/10.3390/xxxxx

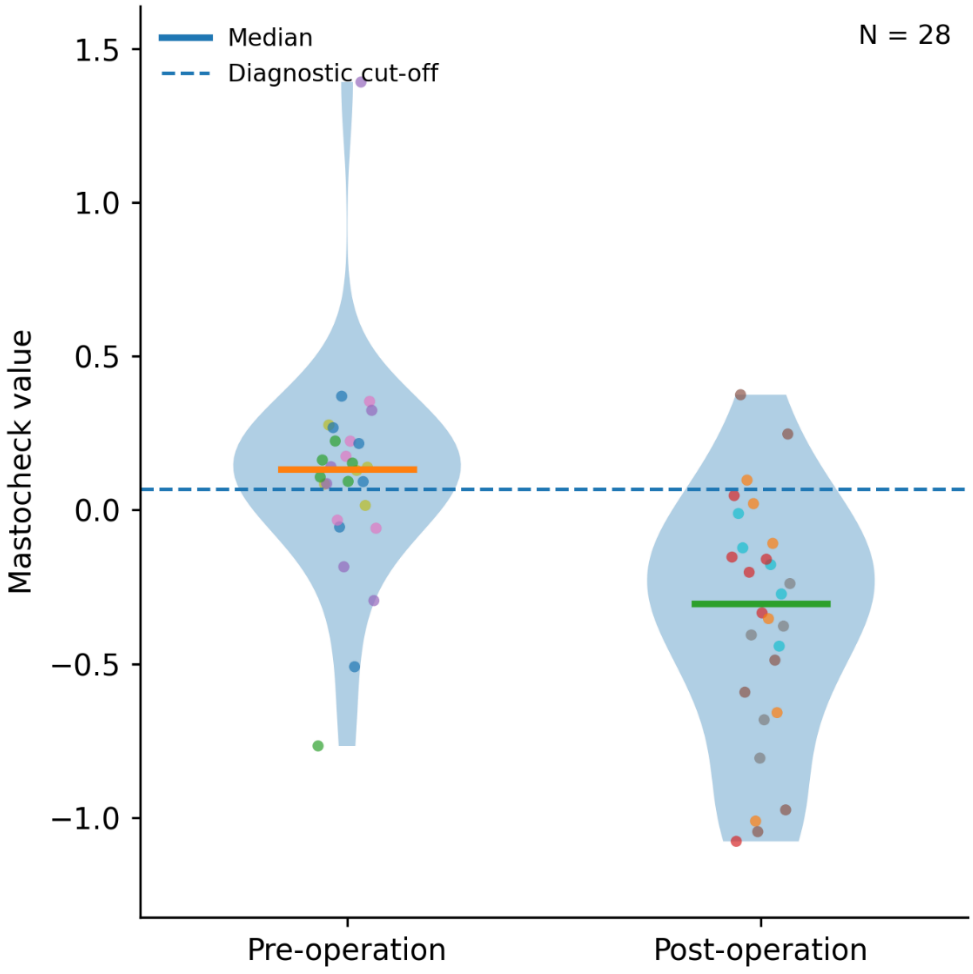

Figure S2. Distribution of Mastocheck values before and after surgery. Violin plots illustrate the distribution of Mastocheck values at the pre-operation and post-operation time points, with overlaid jittered dots representing individual patients (n = 28). Horizontal bars indicate median values. The dashed line denotes the predefined diagnostic cut-off (0.0668). Mastocheck values showed a significant downward shift after surgery, with the median decreasing from 0.134 at pre-operation to −0.304 at post-operation (Wilcoxon matched-pairs signed-rank test, one-tailed [pre-operation > post-operation], $p < 0.001$($p = 8.61 \times 10^{-5}$)).

https://doi.org/10.3390/xxxxx
